# Recurrent Bell’s Palsy During Takeoff on a Commercial Flight: A Case Report

**DOI:** 10.5811/cpcem.2020.12.50340

**Published:** 2021-01-16

**Authors:** Gayle Galletta

**Affiliations:** University of Massachusetts, Department of Emergency Medicine, Worcester, Massachusetts

**Keywords:** Case report, recurrent Bell’s palsy, transient facial nerve palsy, in-flight emergency, altitude

## Abstract

**Introduction:**

Unilateral facial weakness is a concerning symptom, particularly in a resources poor setting. Distinguishing between peripheral and central causes is critical to the evaluation, treatment, and prognosis.

**Case report:**

An unusual case of recurrent, transient Bell’s palsy occurring during ascent in a commercial airplane is presented.

**Conclusion:**

Emergency physicians should be aware of the possibility of barotrauma to the facial nerve (cranial nerve VII) during flights because accurately diagnosing this condition can prevent costly aircraft diversion, calm the passenger’s anxiety, and forgo an expensive medical workup.

## INTRODUCTION

Unilateral facial paralysis may have a central or peripheral etiology. It is important to differentiate between the two because the workup, treatment, and prognosis differ. Transient facial paralysis from peripheral facial nerve paresis (Bell’s palsy) is common, accounting for 60–75% of unilateral facial weakness and has an incidence of 20–30 cases per 100,000 annually.^1^ It can occur at any age but is most common in the fifth decade. Males and females are equally affected as is the laterality.^2^ Most cases of Bell’s palsy resolve completely by six months. Bell’s palsy has been associated with diabetes, hypertension, pregnancy, Lyme disease, herpes simplex type 1 infection, sarcoidosis, and with the inactivated intranasal influenza vaccine.^3^

Bell’s palsy has also been reported to occur from barotrauma, most commonly scuba diving. Less commonly, barotrauma has also been noted to occur with aviation and ascent to high altitude.^4^ Bell’s palsy during aviation was first described in 1967.^4^ Since that time, several case reports have been published, primarily in the otolaryngology literature.^4^–^11^ Recurrent Bell’s palsy is rare, occurring in approximately 7% of cases,^12^ and has only been reported three times during flights,^4^–^6^ but these cases were not published in the emergency medicine literature.

## CASE REPORT

A 36-year-old Dutch Caucasian male without significant past medical history developed a right facial paralysis shortly after takeoff during a transatlantic flight that departed from the East Coast of the United States. A flight attendant was notified and subsequently called for a doctor onboard. The passenger was standing in the rear of the aircraft with the flight attendant. His physical examination revealed that he was anxious, but otherwise in no acute distress. He was not diaphoretic. His heart rate and respiratory effort were normal. His pupils were equal, round, and reactive to light. He exhibited a right facial droop that involved the forehead. His cranial nerves were otherwise intact. There was no extremity weakness. Cerebellar functions were intact and his gait was normal. His speech was normal. Other than the facial droop and the anxiety that this caused, the patient had no other complaints. He denied having a headache or ear pain.

Interestingly, the passenger stated that he had an identical episode shortly after take-off four days prior. The flight was diverted back to its originating airport due to concern for a stroke. The passenger was taken to a hospital, evaluated for a stroke, and admitted overnight for observation. He stated that his work-up was completely normal, but he did not recall whether he had been tested for Lyme disease. He was medically cleared to fly home to Europe on the date of this recurrent episode.

Based on the passenger’s physical examination that included paralysis of the forehead muscles, and further reassured by his recent negative work-up for an identical episode, it was apparent that his symptoms were due to a peripheral nerve etiology rather than a stroke, and the flight would be able to continue to its destination as scheduled. In-flight medical control was not contacted. Shortly after reaching cruising altitude, the passenger’s symptoms resolved.

## DISCUSSION

The anatomy of the facial nerve and the unilateral facial paralysis associated with its disruption was first described by Sir Charles Bell in 1821.^13^ The facial nerve has a primarily motor function and originates in the pons. It travels a long, circuitous route and exits the skull through the internal auditory canal. It then traverses the facial canal adjacent to the middle ear. It has been suggested that temporal bone dehiscence in the tympanic segment of the canal at the oval window can predispose patients to barotrauma of the facial nerve.^14^

Central pathology (stroke, tumor, multiple sclerosis) vs peripheral pathology (Lyme, otitis media, viral, idiopathic) of the facial nerve can often be deduced from the physical exam. Somewhat counterintuitively, a peripheral lesion presents with more extensive facial paralysis that involves the entire hemiface. A central lesion will spare the forehead. This can be explained by the fact that the muscles of the upper face are innervated by the corticobulbar tract bilaterally, whereas the muscles of the lower face are innervated only by the contralateral motor cortex. There are always exceptions, however. Bell’s palsy can occasionally occur bilaterally, and a brainstem stroke at the facial nerve nucleus could mimic Bell’s palsy.^15^

While the etiology is unclear, it is possible that the passenger described above had a predisposing abnormality such as a dehiscence of the temporal bone in the region of the facial canal where the facial nerve courses near the middle ear. Such an abnormality would subject the nerve to temporary ischemia from barotrauma due to the decreased partial pressure of oxygen at decreasing atmospheric pressure during flight ascent.

## CONCLUSION

Diverting a transatlantic flight can cost upwards of 100,000 US dollars. The cost of flight diversion is greatly variable and depends on the size of the aircraft, the cost of dumping fuel, housing and re-booking passengers, and staffing of flight crew. It is important to be aware of the potential for a benign peripheral facial nerve paralysis from barotrauma during flight. Being able to distinguish this from a central stroke (that may benefit from immediate medical treatment) could prevent costly flight diversion and calm passenger anxiety. It would also be helpful for the passenger to understand that their facial nerve paralysis may recur on future flights and resolve without intervention.

**CPC-EM Capsule**

What do we already know about this clinical entity?

The facial nerve can be sensitive to pressure changes such as scuba diving and aviation, resulting in Bell’s Palsy.

What makes this presentation of disease reportable?

Only three reports of transient, recurrent facial nerve paralysis from aviation barotrauma have been published: none in the emergency medicine literature.

What is the major learning point?

Recurrent Bell’s palsy is rare, but can occur in individuals susceptible to changes in ambient pressure.

How might this improve emergency medicine practice?

Distinguishing a benign facial palsy from a more serious etiology in a low resource setting, such as during a commercial flight, could prevent costly flight diversion and calm passenger anxiety.
